# Singling out the double effect – some further comment

**DOI:** 10.1080/17571472.2015.1082346

**Published:** 2015-09-28

**Authors:** Andrew Papanikitas, John Spicer

**Affiliations:** ^a^ Nuffield Department of Primary Care Health Sciences, University of Oxford, New Radcliffe House, Radcliffe Observatory Quarter, Woodstock Road, OxfordOX2 6GG, UK; ^b^ Primary Care Education and Development, Health Education South London, London, UK

**Keywords:** Conscience, ethics, moral, law, contraception, sexual health advice, minor, child, double effect

## Abstract

We comment on a paper published in the same issue of the London Journal of Primary Care. We applaud Bow’s engagement with the ethical issues in a previous LJPC paper but argue that further work is needed to establish the everyday moral concerns of health care workers in primary care. We also suggest that the ethical distinction between advice and medication and devices may be artificial if both have an effect on a patient.

## Key messages

• Further work is needed to establish the everyday moral concerns of health care workers in primary care.
• The ethical distinction between advice and medication and devices may be artificial if both have an effect on a patient.

We are delighted to see a response to an LJPC article [1] by Stephen Bow [2] that engages with the arguments presented. We encourage articulate responses to all LJPC papers. Some of the arguments used by Bow to contest the paper are themselves contestable, and we will briefly raise them here to inform future debate and future lines of research.

There is a discussion to be had about whether sexual health workers who object to the use of various forms of contraception in and of themselves should have a right of conscientious objection. The point has controversially been raised for doctors by Savulescu.[3] Further empirical work is needed to determine how much of an issue this is, for what proportion of clinicians who give sexual health advice and contraception, and how this may become problematic for those clinicians.

There is some philosophical debate over whether pregnancy can be considered to be harmful. Bow is quite right to suggest that many of the arguments that lead this way are consequentialist or utilitarian – arguments range from a threat to the mother’s physical health, through to a societal concern with prevention of births into adverse circumstances. As Bow rightly suggests, societal interests may well be at issue rather than those of the sexually active child.

We think that the distinction between advice and treatment as ‘words’ and a ‘treatment or device’ is possibly artificial. The prescription of condoms might well deter inappropriate sexual activity. Conversely, advice on methods of contraception is arguable contraceptive if it leads to behaviours that prevent conception. A more defensible ethical distinction might be between treatment that prevents conception and treatment that prevents implantation or induces miscarriage – but this is not the discussion at hand in either Papanikitas or Bow papers.

We welcome correspondence regarding the above papers via the LJPC forum.
